# Evaluation of the Sublay Mesh Repair Outcomes in Different Types of Ventral Hernia

**DOI:** 10.7759/cureus.20590

**Published:** 2021-12-21

**Authors:** Mohamed Issa, Khaled Noureldin, Abdelhamed Elgadi, Ayyat Abdelaziz, Marwa Badawi, Mahmoud Makram

**Affiliations:** 1 Surgery, Wirral University Teaching Hospital, Wirral, GBR; 2 Surgery, Prince Charles Hospital, Myrther Tydfil, GBR; 3 General Surgery, Cairo University Hospital, Cairo, EGY; 4 Colorectal Surgery, Southend University Hospital NHS Foundation Trust, Essex, GBR; 5 Vascular Surgery, Benghazi Medical Center, Benghazi, LBY; 6 Obstetrics and Gynecology, Minia University, Minya, EGY; 7 General Surgery, Conquest Hospital, Hastings, GBR; 8 General Surgery, Cairo University, Cairo, EGY

**Keywords:** mesh repair, postoperative complications, recurrence rate, component separation technique, sublay meshplasty, incisional ventral hernia, abdomen ventral hernia

## Abstract

Introduction: Ventral hernia repair is one of the challenging surgical operations over time. Several surgical techniques for mesh repair have been described (onlay, inlay, sublay, and underlay repairs). It is suggested that sublay mesh repair has the lowest recurrence and surgical site infection in open anterior abdominal hernia repair. This study aimed to analyze the pros and cons of the sublay mesh in ventral hernia repair to evaluate the significance of this technique as a treatment modality. Hospital stay, acute postoperative complications, and the recurrence rate were the main areas of investigation.

Methods: A retrospective study on 79 patients with ventral hernias who were operated on with sublay mesh repair between January 2015 and December 2018 was conducted. Patients were admitted through the elective route. The study included fit patients with first-time ventral hernias (primary and incisional). Recurrent hernia, patients with decompensated cardiopulmonary disorders, and bleeding disorders were excluded from the project. The project pro forma includes patient’s demographics, operative details, length of stay, postoperative complications, and follow-up up to 12 months.

Results: All patients underwent open mesh repair using the sublay technique. The ventral hernia was five times more common in females than males. The mean age of presentation was 44.8 years old. The mean operating time was 67 minutes and a one-day hospital stay. Paraumblical and incisional hernias represented the majority of cases. The component separation approach was added in three cases (3.7%). Simultaneous cholecystectomy was performed in two cases (2.5%). Only six cases (6.3%) developed wound-related complications, while two cases (2.5%) had a recurrence.

Conclusion: The sublay mesh repair is a perfect choice for the repair of ventral abdominal hernia. It is associated with a smooth and short hospital stay and the least incidence of complications and recurrence.

## Introduction

Ventral hernias refer to the bulge of abdominal organs through a defect in its anterior wall. This is subdivided into two main groups (pathological and iatrogenic). Incisional hernia post abdominal surgery is the most common complication after laparotomy, with an incidence rate between 2% and 20% [1,2].

The anterior abdominal wall anatomy is composed of skin and subcutaneous fat, followed by Scarpa's and Camper's fascia, while deep fascia is absent from that complex of tissues. Deeper layers include abdominal wall muscles, fascia transversalis, preperitoneal fat, and peritoneum. Above the arcuate line (midpoint between the umbilicus and symphysis pubis), the internal oblique aponeurosis envelops the rectus muscle. The external oblique aponeurosis always sits anterior, while the transversus abdominis lies posteriorly. Below the arcuate line, all muscles become anterior to the rectus abdominis. The two-sided rectus sheath fuses at the midline to form the linea alba and laterally to form the linea semilunaris [3-5].

Long-standing increased intraabdominal pressure (e.g., strain, constipation, chronic cough) weakens the local tissues and leads to microscopic tears, predisposing to hernia formation. Post abdominal surgeries, the tissue strength can only regain 80% of its maximum tensile power. This 80% predicted tensile strength is under perfect conditions, assuming no evidence of malnutrition or infectious complications. Thus, each abdominal surgery is a predisposing factor for incisional hernia [4,5].

Surgical repair is the gold standard line of treatment in a fit patient. About 350,000 cases are treated per year in the United States. The repair of the abdominal wall defect has evolved over time. Before 1993, all ventral hernias were fixed through the open technique. While primary closure of the defect, using sutures, remains the oldest method, it has been proven to have a high failure rate (8%-63%). The integration of the mesh in hernia repair has markedly flattened the recurrence curve (1%-4%). Taking advantage of Laplace's law, the mesh aims to reinforce or bridge the defect and distribute the intraabdominal pressure across the wide synthetic sheet and not just the defect [6,7].

The two popular approaches for positioning the mesh are either onlay or sublay. Each method has its pros and cons. Some surgeons outweigh the onlay technique to reduce extensive dissection. Thus, it is a faster and easier technique; however, local wound complications are high. Therefore, it is believed that the sublay repair has a better outcome. In the sublay approach, the surgeon can place the mesh posterior to the fascial plane. This can be retrorectus, preperitoneal, or intraperitoneal. The latter technique is gaining popularity, accredited to key-hole surgery [8-10].

This retrospective study aimed to evaluate the outcome of the sublay mesh repair for ventral hernias. The study analyzed the operative time, postoperative recovery time, incidence and types of postoperative complications, and, finally, the recurrence rate.

## Materials and methods

This retrospective study was done in Cairo University Hospital, Cairo, Egypt, (Misr International Hospital) from January 2015 to December 2018. It included 79 patients (24-65 years old) who had their operation by four general surgeons. The study's inclusion criteria were uncomplicated types of ventral hernia, which were not subjected to a previous repair.

The following subgroup of patients was excluded: (1) complicated ventral hernias (inflamed, obstructed, or strangulated), (2) recurrent ventral hernia, and (3) patients with comorbidities (uncompensated heart or lung diseases and hemorrhagic disorders) that may affect the repair outcomes.

As a retrospective non-interventional review, this study was locally approved by the Misr International Hospital Ethical Committee to review the patients’ data saved in the hospital records. Clinical letters, admissions, operative data, and postoperative complications were revised. All patients were subjected preoperatively to the following: (1) A detailed history taking and thorough clinical examination. (2) Routine preoperative laboratory investigations, including full blood count (FBC), coagulation profile, random blood sugar, liver, and kidney function tests. (3) Radiological investigations included (a) routine abdominal ultrasonography to exclude any intraabdominal pathology; (b) limited use of abdominal computed tomography (CT) to cases with a huge defect for anterior abdominal configuration; and (C) chest plain x-ray for patients with a history of smoking, bronchial asthma, or clinical signs of chest troubles. Electrocardiography (ECG) and echocardiography were routinely ordered in any patient above 40 years of age.

Surgical technique

**Figure 1:**
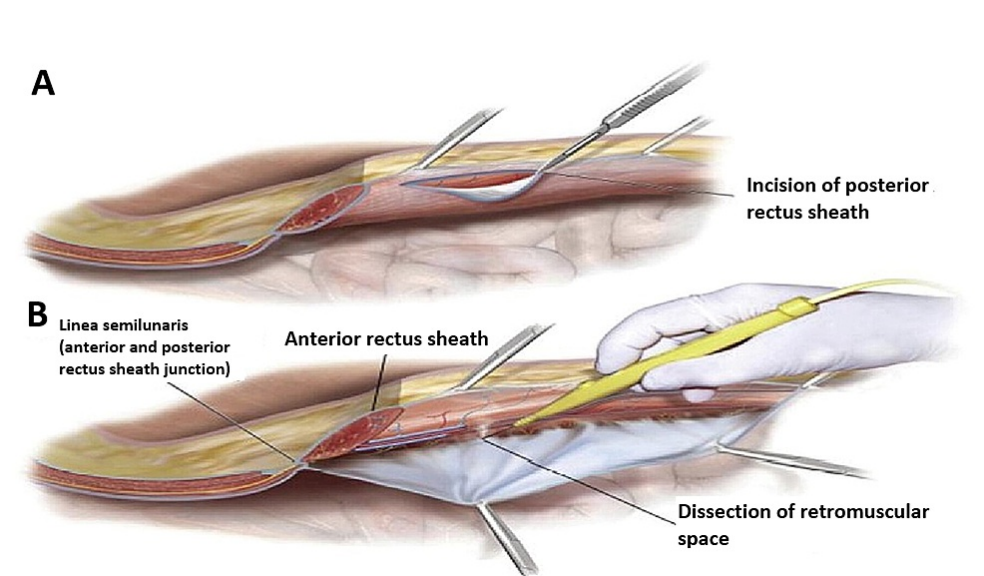
Level of dissection above posterior rectus sheath

The retrorectus or sublay mesh repair principles included two main steps: (1) mesh placement deep into the recti muscles and (2) mesh extension beyond the hernia defect [11]. Typically, the surgery begins with a midline incision carried down through the subcutaneous tissues until the anterior fascia is reached. The hernia sac, which is lined by the peritoneum, is identified, and the fascia superior or inferior to the defect is entered. This fascial incision is lengthened to ensure that there is adequate exposure of the hernia defect. After careful dissection of the hernia sac away from the surrounding fascia, the peritoneum is incised carefully. At this point, a full exploration of the abdomen can take place, and any concomitant procedures may be performed. The medial edge of each rectus muscle was identified by palpation, and the extreme medial edge of each rectus sheath was incised along its length to reach the submuscular space. This relatively bloodless plane could be created to the lateral edges of the rectus muscle on each side. Primary "peritoneal" closure was obtained using posterior rectus sheath above the arcuate line, the peritoneum itself, or excess sac below the arcuate line as shown in Figure 1 (Panels A and B) [12].

**Figure 2:**
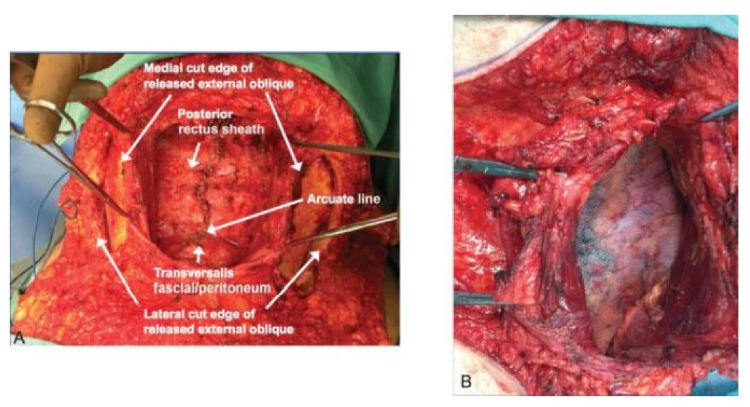
Closure of the posterior rectus sheath and mesh positioning

The posterior rectus sheath, along with the peritoneum, is closed with 2/0 polydioxanone (PDS) sutures. The longitudinal axis of the defect was measured in centimeters, using an intraoperative sterile ruler to determine the suitable size of the mesh. Then, a propyl prolene mesh is fastened around and well beyond the defect (about at least 5 cm) and fixed using prolene 2/0 sutures. The edges of the muscular sheath were sutured over the mesh by nonabsorbable (prolene 2/0) sutures. Drain is left only in cases that need component separation, as shown in Figure 2 [12]. In patients with post appendicectomy incisional hernia, the repair was approached locally through the previous McBurney's incision. All cases have the same day discharge; however, patients with component separation were discharged on the fifth postoperative day.

## Results

A total of 79 patients underwent sublay mesh repair for various types of ventral hernia (January 2015 to December 2018) and were followed up for 12 months. The mean age was 44.8 (range 24-65) years, and the mean defect size was 5.55 (range 3-10) cms. The incidence of ventral hernia was highest in the fourth and fifth decades of life (Table 1).

**Table 1:** Age distribution

Age	Frequency	Percentage
24-34	13	16.5%
35-44	26	33%
45-54	30	35%
55-64	8	10%
65-74	2	2.5%

**Figure 3:**
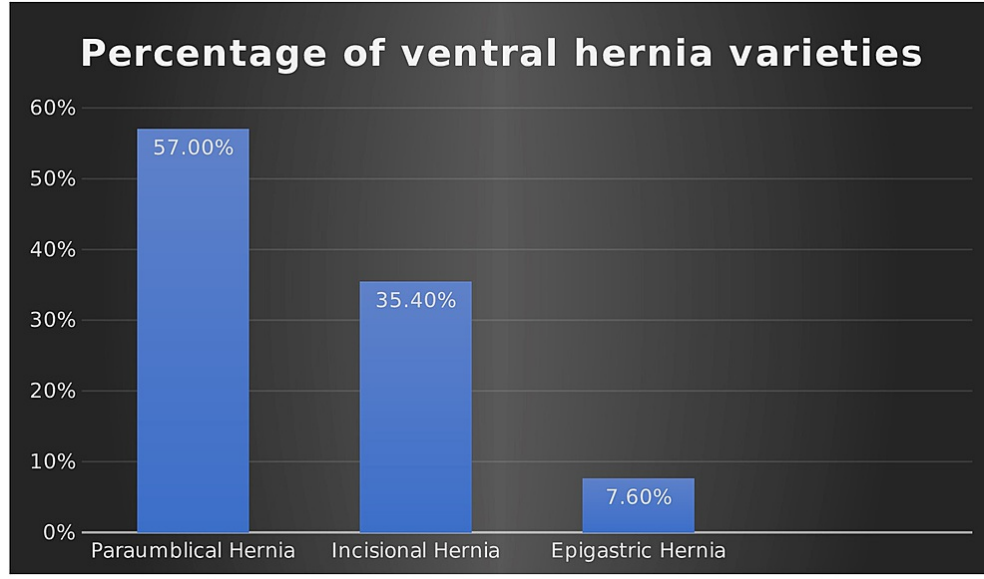
Percentage of ventral hernia varieties

Out of 79 patients, the female to male ratio was 5:1 (66 [83.5%] and 13 [16.5%] retrospectively), showing that the incidence of ventral hernia is higher in females. The main presentation was an anterior abdominal swelling. Seventy-six patients (96%) showed hernia reducibility, while the rest were partially reducible with a preserved impulse on cough; 57% of all ventral hernias were paraumbilical hernia, followed by incisional and epigastric hernias - 35.4% and 7.6%, respectively. There was no umbilical hernia or Spigelian hernia seen in this cohort of patients (Figure 3).

Incisional hernias were secondary to midline incision in nine cases, cesarean sections in six patients, and appendicectomies in two cases. One case had a combined post-cesarean incisional hernia plus paraumbilical hernia, and they were repaired simultaneously.

The mean operative time was 67.093 (range 50-81.5) minutes. The prolonged operative time was due to difficult dissection in obese patients, extensive adhesions, multiple defects, and patients with double pathology. Component separation technique was performed in three cases, and the posterior method, the anterior in another, and the combined approach were performed in the third case. Simultaneous cholecystectomy was undergone in two cases, presented with gallbladder stones, without any intraoperative complications.

Unfortunately, the transverse colon was injured during sac dissection in one case; the small cut was primarily repaired and did not hinder the mesh fixation forward to sublay without further complications.

**Figure 4:**
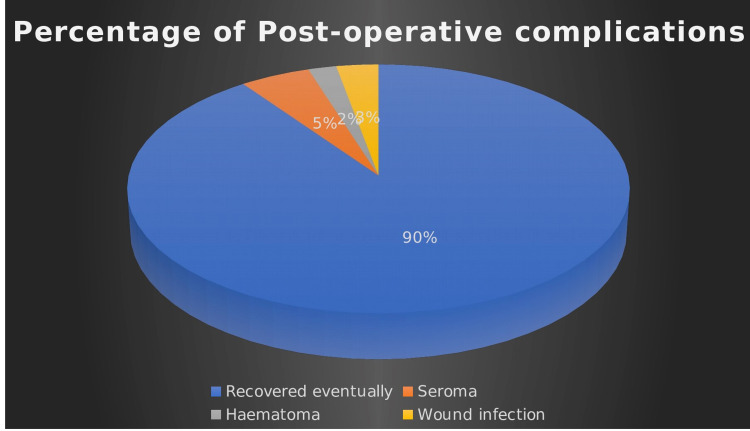
Postoperative complications

During the follow-up period (one year time), 71 patients (89.9%) recovered uneventfully, while eight patients (10.1%) developed various wound-related complications. Two cases developed rectus sheath hematoma (2.5%), and four cases had wound seroma (5%). These cases were observed until complete resolution in six weeks; no aspiration or intervention was done. Deep wound infection was noted in two cases (2.5%), which required antibiotics, drainage, and daily dressing over three weeks. Recurrence of the hernia was witnessed in two cases (4%) as a long-term outcome (Figure 4).

## Discussion

Anterior abdominal wall hernias can be primary (congenital) or secondary (acquired). Two-thirds of the ventral hernias are considered primary, and these can be either congenital or pathological. These hernias are named after their anatomical defects; epigastric (25%), umbilical region (71%), and others (4%). Rare ventral hernias include Spagellian and lumbar hernias. On the other hand, incisional hernias can occur following any abdominal intervention. It is the most frequent withdrawal post midline incisions. Five percent of minimally invasive surgeries may result in port site hernia, while 30% of stoma procedures can develop parastomal hernia [13].

The European Hernia Society (EHS) set a system to classify ventral hernias. The system subdivides these hernias into primary and incisional. A hernia not associated with a previous operation is named primary, subdivided into the midline and lateral, with two variables (length and width). The subtypes of incisional abdominal wall hernias are more sophisticated than primary hernias. They can develop anywhere on the abdomen; they are documented in terms of length and width. The limitation of the EHS system is that it does not involve patients' risk factors and wound classification. However, a classification complex enough to include all of the crucial variables would be difficult and unlikely to embrace the surgical community. Loss of domain term is used when a significant amount (half of the abdominal contents) of the visceral contents are in the hernia sac [14].

Like all types of hernias, the clinical presentations can be classified into uncomplicated and complicated hernias. The uncomplicated hernia will present as a swelling through the abdominal wall, reducible or partly reducible with a preserved impulse on cough. Complicated ones can be inflammation, incarceration, obstruction, strangulation, or perforation of the content; in that case, there will be local and systemic manifestations. In most cases, the diagnosis is usually made through history taking as well as local and general clinical signs. Nevertheless, the patient should be examined in different positions to elicit more signs for better assessment [15,16].

In some instances, adjuncts such as CT scan, abdominal ultrasound, and CT scan volumetry should be utilized to reach a proper diagnosis, rule out underlying etiology (malignancies), and plan the surgical intervention. CT scan of the chest, abdomen, and pelvis helps to rule out the underlying chest and abdominal causes. It also can be used to find strangled viscus and obstructed hernias. Thus, these findings will guide the surgeon for the urgency of the intervention and plan the repair approach. CT scan volumetry can be utilized in complex ventral hernias. It can measure the size of the defect (>10 cm) and assess the ratio between the hernia sac volume to the abdominal cavity volume. Other diagnostic tools can include ECG, echo, chest x-ray, transrectal ultrasound, and endoscopies to investigate the possible causes of the hernias and exceptional cases such as searching for recurrence and distant spread in case of post exploration incisional hernias [16].

Over decades, the surgical approach to repair has been evolved. The key factor is tension-free reconstruction. The most simple technique is primary closure when the defect is less than 2 cm in width. At the same time, other surgical schools prefer the application of synthetic mesh with 3-5 cm overlapping the defect area. Laparoscopic repair is found to have better outcomes when it is compared to the conventional method. It has enhanced postoperative stay and recovery, with extremely low incidences of complications. Some literature has indicated that the recurrence rate is slightly lower in the minimally invasive technique; however, this is not consistently statistically significant. On the negative aspect, laparoscopy has a higher risk for visceral injury, and it is technically more difficult than conventional repair. There has been the development of its ergonomics to increase the freedom of motion. Robotic ventral hernia repairs have also become popular. Closing the fascial defect robotically is far easier and more tempting than using classical laparoscopic instruments [17,18].

This retrospective study investigated the outcomes of the sublay technique to repair a ventral hernia on a sample size of 79 patients. The parameters examined were the mean operative time, postoperative wound complications, and the rate of recurrence. A comparison was made with a two-year prospective study on 102 cases of different ventral hernias. Furat et al. [19] looked at the same parameters while comparing onlay and sublay techniques. Sublay repair was done in 52 patients, which showed almost the same results as this study. According to Furat’s analysis, the operative time was between 65 and 120 minutes; this range was lowered to 50-81.5 minutes in our study. The incidence of seroma was 2% and 5% in Furat’s and our cohort of patients, respectively. Both studies showed equal rates of deep wound infection (2%). While this retrospective study showed two cases of recurrence (4%), the prospective study did not have any recurrent hernia. On the other hand, these figures were higher when Furat et al. assessed the onlay approach on 50 patients. The recurrence and wound infection rates were 2% and 4% retrospectively, while the percentage of seroma rocketed to 24%. 

Another study was run by Shekhar et al. on 100 patients to compare the onlay and sublay mesh repair for ventral incisional hernia [20]. Sublay approach was adopted in 50 cases. Their mean operative time was 55.28 minutes, while it was 67.093 minutes in this cohort research. Shekhar et al. did not report any recurrence or wound infection for the sublay mesh repair. In contrast, they documented 2% and 4% for recurrence and local wound infection, respectively, after one month of follow-up for the onlay mesh-repaired 50 cases. To sum up, according to the previous research work, it is found that the rate of adverse outcomes has favored the utilization of the sublay mesh approach over the onlay method to seek better results.

Timmermans et al. worked on a meta-analysis to compare the outcomes between the sublay and onlay methods for incisional hernia repair; it favored the sublay approach due to lower rates of recurrence and surgical site infections. This was supported by analytic input from the Danish Hernia registry. The data show that the rate of postoperative infections following the onlay mesh repair ranged between 5% and 75% (mean value of 33.5%). These numbers dropped from 8% to 26% (mean value 18.6%) when the sublay mesh repair was used. As for recurrence incidence, the figures were not statistically significant. Onlay mesh had a rate between 0% and 32%, with a mean value of 9.9%. These numbers ranged from 1.6% and 32% (mean value of 13.5%) for the sublay technique. Thus, the main comparison between the two repairs is the postoperative complications [21].

A limitation in this study was not using laparoscopy as a modality to fix the ventral hernia. Thus, we looked at the outcomes from other studies, which included the minimally invasive technique. Orenstien et al. looked up the same parameters that this study looked at. Orenstien's retrospective study included 47 patients in which two patients required endoscopic component separation. The mean operative length was 134 minutes, and the postoperative stay was between 0 and 10 days (average is 2.9 days). They recorded zero intraoperative complications. Although there were no surgical site complications in that study, two major adverse incidences occurred: one case of pulmonary embolism and one case of postoperative stroke. The outpatient follow-up phase was 18 months, and they did not document any recurrence case [22].

## Conclusions

According to this center experience, sublay mesh repair for ventral hernia has favorable outcomes regarding short operative time and hospital stay. Thus, it fits with the enhanced recovery protocol and saves unnecessary hospital costs. It is associated with meager rates of postoperative surgical site complications and recurrence. However, long-term follow-up is recommended to monitor any remote complications. Laparoscopic repair is advised when feasible; therefore, further investigation of its outcomes in ventral hernia repair is recommended.
